# The current progress and future prospects of personalized radiogenomic cancer study

**DOI:** 10.7603/s40681-015-0002-0

**Published:** 2015-02-02

**Authors:** Juhn-Cherng Liu, Wu-Chung Shen, Tzu-Ching Shih, Chia-Wen Tsai, Wen-Shin Chang, Der-Yang Cho, Chang-Hai Tsai, Da-Tian Bau

**Affiliations:** 1 Terry Fox Cancer Research Laboratory, China Medical University Hospital, No. 2, Yuh-Der Road, 404 Taichung, Taiwan; 2 Graduate Institute of Clinical Medical Science, China Medical University, 404 Taichung, Taiwan; 3 Department of Biomedical Imaging and Radiological Science, China Medical University, 404 Taichung, Taiwan

**Keywords:** Radiogenomics;, Cancer study;, Single nucleotide, polymorphism;, Genotype;, Cancer therapy

## Abstract

During the last twenty years, mounting studies have supported the hypothesis that there is a genetic component that plays an important role in clinically observed variability in individual tissue/organ toxicity after radiotherapy. We propose the term “Personalized Radiogenomics” for the translational study of individual genetic variations that may associate with or contribute to the responses of tissues to radiation therapy used in the treatment of all types of cancer. The missions of personalized radiogenomic research are 1) to reveal the related genes, proteins, and biological pathways responsible for non-tumor or tumor tissue toxicity resulting from radiotherapy that could be targeted with radio-sensitizing and/or radio-protective agents, and 2) to identify specific genetic markers that can be used in risk prediction and evaluation models before and after clinical cancer surgery. For the members of the Terry Fox Cancer Research Lab in China Medical University and Hospital, the long-term goal is to develop SNP-based risk models that can be used to stratify patients to more precisely tailored radiotherapy protocols. Worldwide, the field has evolved over the last two decades in parallel with rapid advances in genetic and genomic technology, moving step by step from narrowly focused candidate gene studies to large-scale, collaborative genome-wide association studies. This article will summarize the candidate gene association studies published so far from the Terry Fox Cancer Research Lab as well as worldwide on the risk of radiation-related cancers and highlight some wholegenome association studies showing feasibility in fulfilling the dream of personalized radiogenomic cancer therapy.

## 1. The current status of personalized radiotherapy

The ultimate goal of radiotherapy for cancer is to regain control of the growth of the cancer, and at the same time, minimize the possible adverse effects of the radiotherapy. However, there is no denying that cancer patients receiving radiotherapy alone or in combination with chemotherapy display a lot of individual difference in tissue responses. Even though amazing technological improvements have enabled a more precise focusing and killing of the tumor cells, there is still some normal tissue that is inevitably exposed to radiation and that becomes abnormal [1]. In some cancer survivors, this radiation exposure leads to cytotoxicity, tissue toxicity or organ dysfunction and affects their quality of life. This is of particular importance for those patients diagnosed with early-stage localized cancer with a favorable prognosis who may live for a number of years after cancer therapy in the hospital. With increasing cure rates for common cancers such as nasopharyngeal carcinoma, oral, breast, lung, prostate and cervical cancers, ‘survivorship issues’ are attracting more and more attention and discussion among patients and their relatives [2]. Thus, it is critical for radiologists and doctors to reconsider the morbidity that could result from radiotherapy and the necessary steps that might be undertaken to prevent these complications, as well as to consider offering alternative treatments to patients at the highest risk. The study of the possible links between genomic variations and responses to radiation therapy, radiogenomic cancer study, is a multi-disciplinary and practically translational field of research. If successful, the work would not only lead to strategies that reduce the negative impact of these toxicities for cancer patients, but also lessen the financial burden placed on the health care system to care for people suffering from radiation-induced injuries. Identification of sensitive patients would also permit safe dose escalation in more resistant individuals, leading to an increase in local control and cure for some cancers. Therefore, radiogenomic cancer therapy has the dual potential to both reduce toxicity and to permit dose escalation in personalizing radiotherapy [3].

## 2. The spirits of single nucleotide polymorphisms and genome-wide analysis studies

Single nucleotide polymorphisms (SNPs) account for most of the known genetic variation among individuals and are usually defined as polymorphisms in which the minor variant (allele) is present in at least 1% of an investigated population. Non-synonymous SNPs can affect protein function by altering the amino acid composition or by affecting various aspects of the sequential transcriptional and translational outcomes. However, most SNPs are located in regions without any apparent genes or yet-identified functional elements. About 11.8 million SNPs are now included in the National Center for Biotechnology Information’s public database (www.ncbi.nlm.nih.gov/projects/SNP). Among them, less than half of the SNPs have been validated experimentally, while the rest are yet-unconfirmed variations mainly identified through computational prediction and analysis. SNPs are not uniformly distributed among the 3 billion base pair (bp) human genome and there are more than 10,000,000 of them identified.the average length of a gene is about 27,000 bp. That about 100 SNPs are present nearby or within a typical gene. Most SNPs are dbSNP (about 80%) and are single nucleotide variations, also known as “true” or “typical” SNPs. The remaining SNPs are mainly small insertions/deletions including one or a few nucleotides and this type of SNP is called “INDELs”.

Now we turn to the radiogenomic studies about the annotation for the SNPs. In the following we are going to discuss briefly and concisely the two commonly used methodologies, candidate gene approach and genome-wide analysis study (GWAS)., Initial radiogenomic research focused on candidate gene studies, i.e. studying common SNPs in gene-encoding proteins associated with responses to radiation, such as those in charge of DNA repair and cytoskeleton remodeling. This approach by candidate gene studies has the advantage of a relatively low cost, and it uses a priori knowledge about the biological functions involved in radiation response, thus it limits the scope of studies to a manageable number of investigated genes. Prior to recent improvements in the technology for large-scale genotyping and advances in the field of meta-analysis genetic statistics, this approach allowed oncological scientists to conduct affordable studies with modest sample sizes, often with one thousand individuals or less. For instance, the Terry Fox Cancer Research lab has collected hundreds of lung, liver, gastric, colorectal, renal, bladder and prostate cancer patients in central Taiwan. We have also recruited thousands of patients for the most prevalent cancers we are interested in such as oral and breast cancer. Statistically, significant associations were reported for SNPs in a variety of candidate genes and many of these studies have been reviewed previously [4-12]. These associations have to be replicated in validation studies in both the same ethnicity and others to strengthen their contributions to the whole world, or they will have little value in clinical practice [13]. Part of the reason for the lack of reproducible results for most of the SNPs investigated is due to shortcomings in study design, inclusion and exclusion criteria, and experimental performing progresses such as failure to control for individual ancestry and failure to correct *P*-values for multiple-factor stratification and ad adjustment. These problems may be further compounded by publication bias. From a statistical point of view, the candidate gene approach considerably reduces the dimensionality of the data set and therefore efficiently increases the statistical power with a limited sample size. From a biological point of view, however, it may be too restrictive to search for SNPs only in the relatively limited genes that are widely accepted as being involved in radiation damage induction and processing.

The candidate gene approach has also been used to study the association between genetic variants and disease susceptibility and outcomes. Up to now, the genomics community has recognized several important limitations for this approach. One problem is that many studies assay just one or a few SNPs within a gene of interest, but many genes contain tens to hundreds of common SNPs [14]. While careful study design can take advantage of linkage disequilibrium between SNPs to capture a large proportion of variation within a gene region, most candidate gene studies have not performed this type of analysis. This is not in itself a limitation to the candidate gene approach, but it does reflect how studies have been performed until recently. A more serious biological limitation is that we are only just beginning to understand the effects that SNPs have on gene expression and protein function. It is often the case that some SNPs found to be associated with a disease or phenotype of interest are not themselves protein coding variants, but often candidates for affecting transcriptional regulation of a gene that may not be the nearest in proximity. For instance, the prostate cancer risk-associated SNP rs339331 lies within a HOXB13 transcription factor binding site and affects the expression of a more distant gene, RFX6 [15]. MicroRNAs such as mir27 may affect the expression of HOXA10 [16]. Most of all, many of the SNPs selected for candidate gene studies of cancer risk were not replicated in larger GWAS or other populations [17, 18]. So, while some of the candidate genes themselves proved to be of importance, this limited approach to SNP selection prevented identification of the most important loci. At present, in the radiogenomic candidate gene studies, the SNPs are selected on the basis of changes in the amino acid sequence of the candidate gene, similar to other candidate gene studies. Potentially important SNPs that lie in regulatory regions affecting expression of the candidate genes have likely been missed using this limited approach. Finally, the candidate gene approach relies on the completeness and accuracy of our understanding of the biology behind normal tissue reactions to radiation exposure. Much knowledge of the biological response to radiation comes from cell-based studies, but less is known about what occurs in whole tissues, or interacting tissue systems, upon exposure to radiation.

In response to the lack of success of candidate gene SNP studies and recognition of the above-mentioned limitations inherent in this approach, the focus of radiogenomics has shifted towards GWAS. The Radiogenomics Consortium (RGC) was established [19, 20] to enable the collaboration and data sharing needed to obtain the large sample sizes and multiple studies required for effective GWAS. The RGC is a National Cancer Institute/NIHsupported Cancer Epidemiology Consortium (http://epi.grants.cancer.gov/Consortia/single/rgc.html) that’s overall aim is to provide a structure in which tissue samples and data can be pooled from investigators performing radiogenomics work on a global scale. The RGC has been essential in facilitating a shift in radiogenomics from narrowly focused single center studies to a multiinstitutional approach and from candidate gene studies towards a broader, genome-wide approach.

With the rapid development in the technological and quality controlling systems, arrays in biochips have been made available that allow the simultaneous analysis (genotyping) of hundreds of thousands of SNPs in each sample. Using the available information provided by the Human Genome Project and the customermade service from the genotyping company, it is very convenient now for scientists to design SNP arrays that span the entire genome. The HapMap project describes the statistical relatedness of SNPs by providing a catalog of how SNPs are organized on chromosomes and distributed among different populations (www.hapmap.org). Adjacent SNPs are often linked together into some boxes, and regions of linked variants that are inherited together are known as “haplotypes”. Typically, only about 5 different common haplotypes (with frequencies ~5%) are found in most parts of human genome [21]. Within a given haplotype, it is therefore possible to identify particular variants that can predict or “tag” the presence of particular variants at other sites. These SNPs that can be used to uniquely identify haplotypes are known as “tag” SNPs. A good GWAS researcher has to pay more attention to the selection of the investigated samples, ensure a sufficient sample size, and control the stratification criteria, not just collect more and more funding for their research project. In Taiwan, some clinical researchers are naïve and apply for huge amounts of funding for a project with only the methodology of GWAS for a simple disease. The feasibility of such a project may be enhanced with the monopoly of the specific patients for the disease, but it lacks insight and prospective for the near future.

## 3. The efforts toward revealing cancer susceptibility genotypes for Taiwan cancer patients from the point of view of radiologists

Environmental carcinogens such as radiation may induce DNA single and double strand breaks in the cells and those double strand breaks are a very severe type of DNA damage which should be repaired by the DNA double strand break repair subpathway as soon as possible [22, 23]. Mechanically, if cells cannot remove them immediately by means of homologous recombination (HR) and non-homologous end-joining (NHEJ), those DNA double strand breaks may induce precancerous lesions and cancer itself as well [24, 25]. Thus, it is reasonable to propose a hypothesis that genetic polymorphisms in DNA double strand break repair genes influence DNA repair capacity and confer predisposition to several cancers, including skin [26], breast [4, 27], liver [28], gastric [6] and oral cancer [7, 8]. Because of this, we have devoted ourselves for years to the mission of finding potential cancer biomarkers for Taiwanese cancer patients as the cancer predictive and preventive targets. Please keep in mind that the results of a study with a candidate gene approach may not be as splendid as a GWAS, but the significance may be even more solid and useful in clinical practice. From 2008 to 2011, we were investigating the contribution of X-ray cross-complementing groups 4, 5 and 6 (XRCC4, 5 and 6) genotypes to common cancers in Taiwan. XRCC4 is found to restore DNA double strand break repair and has the ability to support V(D)J recombination of transiently introduced substrates in the XR-1 CHO cell line [29]. The XRCC4 protein interacts directly with Ku70/Ku80, and it is hypothesized that XRCC4 may serve as a flexible tether between Ku70/Ku80 and its associated protein ligase 4 [30]. We have found that those who had G/T or G/G at XRCC4 G-1394T (rs6869366) showed a 3.79-fold (95% confidence interval = 1.47-9.82) increased risk of gastric cancer compared to those with T/T [6]. As for oral cancer, those who had heterozygous del/ins at XRCC4 intron 3 (rs28360071) showed a 1.57-fold (95% confidence interval = 1.12-2.21) increased risk of oral cancer compared to those with ins/ins [7]. The genotypes of the former SNP were also associated with the personal susceptibility for other types of cancer, such as myoma [31], endometriosis [32], childhood leukemia [33], breast [34], prostate [35], bladder [36], lung [37], and colorectal cancer [38]; while the latter was also associated with childhood leukemia [33]. In addition to these two novel SNPs, the XRCC4 codon 247 (rs3734091) was also associated with increased oral cancer risk [39]. As for XRCC5 (also named Ku80), there were significant differences between oral cancer and control groups in the distributions of their genotypes (*P* = 0.0038) and allelic frequencies (*P* = 0.0044) in the Ku80 promoter G-1401T (rs828907) SNP [9]. After the stratification of personal betel nut chewing status, it was found that the G/T plus T/T genotypes at Ku80 promoter G-1401T significantly enhanced the risk only in the areca chewers (odds ratio = 1.603; 95% confidence interval = 1.053- 2.011), not in the non-areca chewers [9]. This risk genotype is also associated with an increased risk of bladder [40], colorectal [41] and breast cancer [42] in Taiwan. Similarly, the genotype of XRCC6 (also named Ku70), could be associated with pterygium risk at T-991C (rs5751129) [43], oral [8], gastric [44], kidney [45], and liver cancer [28] as well as childhood leukemia [46]. As for this important SNP at the promoter region, the genotype-phenotype correlation was proved at transcriptional and translational levels. The results showed that the mRNA and protein expression levels in HCC tissues had significantly lower XRCC6 mRNA and protein expressions in the HCC samples with TC/CC genotypes compared with those with the TT genotype (P = 0.0037 and 0.0003, respectively) [28]. The most valuable literature produced from the candidate gene rationale in our mind is the findings that the A-2841T polymorphism of SLC2A1, one member of the facilitated glucose transporter family, was significantly associated with 2-[fluorine-18]-fluoro-2-deoxy-D-glucose-uptake in combination with the apurinic/apyimidinic endonuclease Asp148Glu (T to G) polymorphism in the squamous cell type of non-small-cell lung cancer [47]. As far as radiologists are concerned, the uptake of ^18^F-deoxyglucose (FDG) of PET, expressed as the SUVmax, is largely dependent on glucose metabolism in lung cancer. SLC2A1 is reported to be the primary glucose transporter of glucose metabolism, and the literature has shown that overexpression of SLC2A1 plays a critical role in the survival and rapid growth of the cancer cells in a suboptimal environment [48]. It is also reported that high FDG uptake is associated with lower overall survival and disease-free survival among non-small cell lung cancer patients [49].

## 4. Other outstanding research, especially those GWAS in radiogenomic cancer research

In the first few years, RGC was successful in enabling radiogenomic researchers to complete and publish several GWAS on radiotherapy toxicity. The first radiogenomic GWAS report was a small pilot study with a sample size of only seventy-nine patients with erectile dysfunction following radiotherapy for prostate cancer [50]. Despite the relatively small sample size, one SNP was identified with a very significant *P*-value (*P* = 5.5*10^-8^) and several others were identified that were suggestive of significance (*P* < 1.0*10^-6^). While this study only included a discovery set, and the SNPs identified must be validated, it highlights the potential of GWAS in surveying genes not previously known to be important for radiotherapy response [50]. The most important SNP identified in this study is located within the FSHR gene, which encodes the follicle stimulating hormone receptor involved in gonad development and function [51]. While it may have been expected to reveal a significant association of those genes involved in DNA damage recognition and/or relevant DNA repair pathways, this study also identified a SNP on a gene involved in normal cellular function. This finding does not mean that those genes known to be involved in routine radiation responses do not play a role in the etiology of radiation-induced toxicities, but suggests that other tissue-specific pathways may also be of some importance and play a role in the radiogenomics. This is evidence that a rough GWAS with less SNPs investigated or too small of a sample size may have lost lots of information and got too many false-positive findings, even with significant *P*-values. This kind of finding, or other studies revealing those unknown SNPs without any gene nearby, may add complexity and provide insight to cancer genomics and radiogenomics.

A second GWAS study in radiogenomics we want to discuss here was another study of prostate cancer patients who were assessed for three toxicity endpoints following radiotherapy: urinary toxicity, erectile dysfunction, and rectal bleeding [52]. This study included a moderate population with about 800 prostate cancer patients, and the dataset was split into discovery and replication sets. For the urinary toxicity and erectile dysfunction endpoints, the top SNPs fell short of genome-wide significance, but for each endpoint, several suggestive loci were identified. An 8-SNP haplotype block was associated with urinary toxicity, in which the top SNP rs17779457 was associated with a 2.7-fold increase on the American Urological Association Symptom Score (AUASS) (95% CI 1.2, 4.1) in the discovery set and a 2.4-fold increase (95% CI 1.1, 3.6) in the replication set (*P*-value = 6.5*10^-7^) [52]. This haplotype block is located within *IFNK*, a member of the type I interferon family of immunological genes in charge of inhibiting IL-12 signaling and modulating the cytokine release from innate immune system cells [53]. SNP rs11648233 was associated with erectile dysfunction (*P*-value=9.1*10^-5^), and this SNP is located within the 17-beta-hydroxysteroid dehydrogenase II gene (*HSD17B2*) that catalyzes the oxidative metabolism of androgens [54]. The top locus associated with rectal bleeding contained two SNPs in linkage-disequilibrium, of which rs7120482 with a minor allele frequency (MAF) of 0.37, approached the strict cut-off for genome-wide significance with a *P*-value of 5.4*10^-8^, and thus represents a promising risk locus [55]. This locus lies upstream of *SLC36A4*, which can modulate the activity of the mammalian target of the rapamycin complex 1 (mTORC1) signaling cascade that affects angiogenesis, proliferation, cell survival, and cellular radiosensitization [56-59]. The study revealed several SNPs associated with intracellular radiosensitivity from the radiogenomic viewpoint of radio-oncologists.

A third GWAS study famous in radiogenomics was about three interesting SNPs among prostate cancer patients, with each of the three being associated with a syndrome of prostate cancer patients. They were associated with decreased urinary stream, rectal incontinence, and increased urinary frequency, respectively [60]. But the findings in the discovery database could not be represented in validate populations. Similarly, in 1,194 breast cancer patients, one SNP neared genome-wide significance in the discovery set for association with telangiectasia but was not replicated in an independent dataset [61]. From this example we know that it is not easy to find a set of conclusive SNPs for either early predictors of radiosensitivity or prognosis outcomes.

In addition to these kinds of GWAS, recent studies have begun to investigate variation in mitochondrial DNA (mtDNA). There are compelling reasons to hypothesize that such variation could be important in radiation responses, including the role that the mitochondria and mtDNA-encoded genes have on apoptosis signaling and generation of reactive oxygen species. Two studies examined the association between mtDNA variation and radiotherapy responses. The first, in 32 nasopharyngeal carcinoma patients treated with radiotherapy, found that individuals with ≥ grade 2 fibrosis had more nonsynonymous mtDNA variants than individuals with ≤ grade 2 fibrosis [61]. This study also identified a significant association between the nonsynonymous A10398G variant in the NADH dehydrogenase subunit 3 gene and fibrosis. A second larger study of 606 prostate cancer patients evaluated for radiotherapy-related urinary and gastrointestinal toxicity, did not find any significant associations among a panel of mtDNA variants, including the A10398G variant [62]. Up to now we know that most of the research is performed among prostate cancer patients since it is the most prevalent cancer among males in the USA. The mtDNA has less DNA repair system proteins than the genome in the nucleus and is possibly associated with most aging diseases including all types of cancer. Of course, further large-scale studies of mtDNA are also in urgent need to provide definitive answers for radiogenomics.

## 5. Future directions for radiogenomic cancer research and therapy

Clinical radiosensitivity represents the response to a fairly welldefined exposure (i.e., radiotherapy), but it is in other respects a complex phenomenon, and future research efforts must be aware of this fact. The normal tissue response to radiotherapy is made up of a number of different distinctive types of normal tissue reactions. It has been a long standing discussion in radiogenomics whether we should expect genetic factors to have a general impact on radiosensitivity across different types of reactions or if the impact would be specific to certain reactions [63]. The GWAS recently published by Barnett et al. may shed light on this question. Even though this study had substantially more power to detect associations for overall toxicity, the strongest associations were in fact shown for individual endpoints [60]. This observation may seem at odds with the fact that most of the rare radiosensitive syndromes with Mendelian inheritance seem to inflict a general enhancement of clinical radioresponsiveness [64]. Nevertheless, the observation is consistent with clinical data indicating that no strong association exists between the risks of developing different types of normal tissue toxicity [65, 66]. Thus, the initial results of GWAS underline the importance of not only looking for associations with regard to overall toxicity but also addressing separate toxicity endpoints. Future efforts should take this into consideration when developing study designs and data analysis plans.

Going forward, three main directions are promising in fulfilling the dream of personalized radiogenomics. The first is to enlarge the sample size and expand the investigated cancer from prostate cancer to others as well. The second is to establish a worldwide bioinformatic databank for annotation of these novel SNPs for radiogenomics. The third is to establish individual cell lines from the patients to investigate their radiosensitivity and redioresponsiveness in order to selectively kill cancer cells while protecting non-tumor cells.

To substantially increase the size and number of studies has been proposed by the OncoArray consortium [67] and cancer genomics researchers from nearly 50 countries have formed a collaborative group that is currently in the process of genotyping over 400,000 DNA samples, of which roughly 200,000 are from people diagnosed with either prostate, breast, lung, colorectal or ovarian cancer, using a custom SNP platform called the OncoArray. This SNP microarray has both a GWAS backbone consisting of 260,000 SNPs plus 440,000 SNPs that have been associated with one or more type of cancer or cancer-related outcome. This includes approximately 2,000 SNPs nominated by investigators for which initial evidence was obtained linking them with variation in toxicity following radiotherapy. This worldwide collaboration will permit a large expansion in sample size for radiogenomic studies and shorten the time required to discover useful radiogenomic biomarkers.

**Fig. 1:**
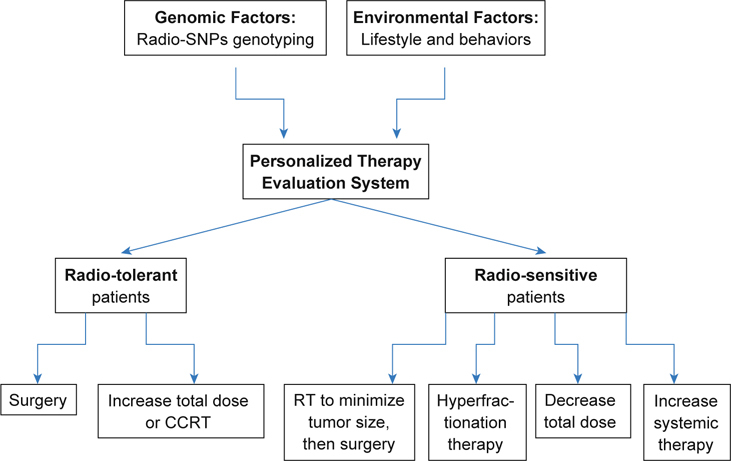
Personalized therapy evaluation system that considers the individual genomic factors, environmental factors, and radiosensitivity for each cancer patient.

As for establishing a worldwide bioinformatic databank for annotation of these novel SNPs for radiogenomics, the participation of nurses, caretakers, patients, and relatives are as important as that of surgeons and scientists. The nurses should record the behaviors and lifestyles with the help of the patients and their relatives, the caretakers should record the responses and adverse effects of the patients to concurrent chemoradiotherapy or radiotherapy with or without nutrition supplements such as AVEMAR.

The last part is the culturing of tumor and non-tumor cells from the patients during surgery and examining their individual sensitivity to those known or unknown combinations of radiation with radiosensitizers or radioprotectors. The ideal protocol for radiotherapy is to kill most of the tumor cells while protecting most of the non-tumor cells. However, the dose of both chemoor radio-therapy should be fine-tuned according to the cells from each patient, and it is not easy to reach such an optimized condition. Only with the establishment of all three parts can we complete the perfect predictive models for personalized radiogenomic cancer therapy. In a perfect model, the records in part I for the genomic factors and in part II for the environmental factors will be used to stratify patients according to their predicted risk level, and then each patient will be arranged to a treatment protocol with the experimental data gathered from part III. For instance, individuals predicted to be at a high risk for developing normal tissue toxicity may experience better outcomes if given a more targeted form of radiotherapy. Alternatively, treatment protocols that avoid radiatherapy, such as the use of surgery or chemotherapy, may be more appropriate for this proportion of cancer patients. For the majority of individuals who are predicted to be at low risk for toxicity, dose escalation protocols could be tested with an aim to improve cancer cell killing rates without increasing toxicity to normal cells. In addition, we anticipate that the genes and pathways uncovered by agnostic approaches to genotyping studies will lead to novel hypotheses regarding the biology of radiation response. Investigations of these hypotheses could lead to the development of molecular interventions for preventing the adverse effects of radiation on normal tissues and organs. A personalized therapy evaluation system is shown in Figure 1.

## 6. Conclusion

We strongly believe that the dream of personalized cancer therapy and medicine will be fulfilled in the near future. Before that, the predictive systems for cancer drug resistance, responses to radiotherapy or radiochemotherapy, and cellular toxicity to radiotherapy or radiochemotherapy should be as widely disseminated and carefully studiedas possible. Although the ongoing development of radiogenomic cancer research is promising, the participation of all of us is needed if we are to end the war against cancer.

### Acknowledgements

This work was supported by the Terry Fox Cancer Research Foundation in China Medical University and Hospital. The authors deeply appreciate the great help in genotyping from the following group of outstanding young scientists from the Department of Biomedical Imaging and Radiological Science at China Medical University: Hong-Xue Ji, Chia-En Miao, Lin-Lin Hou, Tzu-Chia Wang, Yun-Ru Syu.
